# Bolus MPTP Injection in Aged Mice to Mimic Parkinson Disease: Effects of Low-Dose Antioxidant Treatment with Fullerene (C_60_) and Fullerenol (C_60_(OH)_24_)

**DOI:** 10.3390/biomedicines13102425

**Published:** 2025-10-03

**Authors:** Tatyana Strekalova, Alisa Burova, Anna Gorlova, Kirill Chaprov, Anastasia Khizeva, Joana E. Coelho, Evgeniy Svirin, Polina Novikova, Lia Ohanyan, Johannes J. M. P. de Munter, Naira Ayvazyan, Luisa V. Lopes, Aleksei Umriukhin, Gohar Arajyan, Harry W. M. Steinbusch

**Affiliations:** 1 Division of Molecular Psychiatry, Center of Mental Health, University Hospital Würzburg, 97080 Würzburg, Germany; 2 Department of Basic and Biomedical Sciences, Université Paris Cité, 75006 Paris, France; 3 Neuroplast B.V., 6229 EV Maastricht, The Netherlandsh.demunter@neuroplast.com (J.J.M.P.d.M.); 4 Research and Education Resource Center, Peoples Friendship University of Russia (RUDN University), 117198 Moscow, Russia; 5 Institute of Physiologically Active Compounds at Federal Research Center of Problems of Chemical Physics and Medicinal Chemistry, Russian Academy of Sciences, 119071 Chernogolovka, Russianastya.khizeva@mail.ru (A.K.); 6 Faculdade de Medicina de Lisboa, Gulbenkian Institute for Molecular Medicine, 1649-028 Lisbon, Portugal; jfecoelho@medicina.ulisboa.pt (J.E.C.); luisa.vlopes@gimm.pt (L.V.L.); 7 Department of Normal Physiology, Sechenov University, 119991 Moscow, Russia; novikova_p_a1@student.sechenov.ru (P.N.); alum1@yandex.ru (A.U.); 8 Laboratory of Toxinology, Orbely Institute of Physiology of NAS RA, Yerevan 0028, Armenia; ohanyanlia@yandex.ru (L.O.); taipan@ysu.am (N.A.); 9 Pharmacology and Pathohistology laboratory, Scientific Technological Center of Organic and Pharmaceutical Chemistry, The National Academy of Sciences of Armenia, Yerevan 0019, Armenia; arajyankens@gmail.com; 10 Department of Cellular and Translational Neuroscience, Faculty Health, Medicine and Life Sciences, Maastricht University, 6229 HA Maastricht, The Netherlands; h.steinbusch@maastrichtuniversity.nl

**Keywords:** Parkinson’s disease (PD), 1-methyl-4-phenyl-1,2,3,6-tetrahydropyridine (MPTP), dopamine, aging, sex bias, nitric oxide synthase, antioxidants, fullerene, fullerenol, behavior, mice

## Abstract

**Background**: Parkinson’s disease (PD) is a neurodegenerative disorder for which no curative therapies currently exist. Experimental models employing 1-methyl-4-phenyl-1,2,3,6-tetrahydropyridine (MPTP) reproduce PD features such as striatal dopaminergic dysfunction and motor deficits. Various MPTP dosing regimens are used to screen drug candidates for PD, but their validity is limited because of the predominant use of young male animals. Sex bias is another issue that is underrepresented in PD research, since females are more susceptible to this pathology. Here, we studied the model of bolus administration of MPTP (30 mg/kg) in aged female mice and assessed its sensitivity to the antioxidants fullerene C_60_ and fullerenol C_60_(OH)_24_, given that oxidative stress is a key contributor to PD. **Methods**: 12-month-old female C57BL/6 mice received fullerene (0.1 mg/kg/day, via diet) or fullerenol (0.15 mg/kg/day, via drinking water). On day 10, mice were injected with MPTP. We studied tremor, piloerection, and behavior in the pole test, rotarod, pole test, and open field. High-performance liquid chromatography (HPLC) was employed to study dopaminergic neurotransmission, and the expression levels of its molecular regulators and nitric oxide synthase (NOS)-related targets were investigated using RT-PCR in the striatum and cortex. **Results**: MPTP-challenged mice displayed profound impairment in markers of dopaminergic neurotransmission and cellular distress, and showed disrupted motor behavior and vegetative functions. Antioxidant-treated animals that received a bolus injection of MPTP demonstrated partial preservation of tremor response, dopaminergic parameters, and *iNOS* and *nNOS* gene expression, although motor performance in the pole test was only modestly improved. Fullerenol appeared more effective in decreasing MPTP-induced neurochemical changes. **Conclusions**: The applied MPTP model showed its validity in mimicking PD features and was sensitive to low doses of antioxidants, suggesting its usefulness for screening drugs that target oxidative and nitrosative stress. The neuroprotective effects of fullerene-based compounds suggest their potential utility in the treatment of PD.

## 1. Introduction

Parkinson’s disease (PD) is a progressive neurodegenerative disorder characterized by the selective loss of dopaminergic neurons in the substantia nigra pars compacta, resulting in striatal dopamine depletion and the development of tremors, bradykinesia, rigidity, and postural instability [1]. In addition to motor symptoms, patients frequently develop a spectrum of vegetative and behavioral non-motor symptoms, all of which contribute to disability and a reduced quality of life [2]. PD remains the second most common neurodegenerative disorder associated with aging and affects approximately 1% of the population [3]. Levodopa remains the most commonly used therapy for alleviating motor symptoms in PD [4]. However, levodopa-based pharmacotherapy causes serious side effects in patients with PD, including dyskinesia, nausea, vomiting, and orthostatic hypotension [5]. These adverse events also severely affect the quality of life of PD patients and reduce treatment adherence, thus necessitating the urgent need for better-tolerated therapeutic strategies [6,7,8]. This requires extensive drug research and development that is reliant on the effective screening of drug candidates for PD treatment [9].

As such, the use of animal models is indispensable in this aspect [10,11]. Currently, genetic models [12], toxin-based paradigms using 6-hydroxydopamine [13], rotenone [14,15], paraquat [16], and reserpine [17], as well as central administration of α-synuclein fibrils [18] and viral vectors [19] have been used to induce PD-like symptoms in rats and mice, among other paradigms [10]. Among PD models using toxins, the administration of 1-methyl-4-phenyl-1,2,3,6-tetrahydropyridine (MPTP) to small rodents became broadly exploited, due to the capacity of this chemical to induce the key pathophysiological PD elements—striatal dopaminergic neurodegeneration, mitochondrial impairment, and oxidative stress [11,20,21].

One of the advantages of MPTP-based rodent models of PD is that they recapitulate the oxidative stress processes associated with this disease and thus show sensitivity to antioxidants [22], which is a potentially promising basis for future pharmacotherapy of PD [23,24]. Most PD models commonly employ young male rodents [25,26]. This seriously limits the validity of PD paradigms as aging-related mechanisms are crucial in PD biology [27,28]. For instance, Battaglia et al. used adult male mice and observed moderate dopaminergic disruption without any marketable neurodegeneration [29]. As such, repeated MPTP dosing is required in young male rodents [25].

Sex bias is also known to be important for PD, as women often show greater vulnerability to this disease, distinct symptoms, and therapy response profiles [30,31]. Young female C57BL/6 mice exhibited greater dopamine loss than males following MPTP administration in a subacute model involving four intraperitoneal injections of 20 mg/kg MPTP over 24 h [32]. Antzoulatos et al. reported that female mice were significantly more vulnerable than males, e.g., when a single 30 mg/kg dose of MPTP was administered to middle-aged animals [33].

Available modifications of the MPTP model are based on intraperitoneal, subcutaneous methods of MPTP administration that can be chronic, subchronic, acute, or bolus (see Supplementary Table S1) [11,34]. Distinct protocols may determine the nature of cell death, as well as the involvement of inflammatory processes [29,35]. Chronic and subchronic models based on repeated or long-term low-dose administration of MPTP to animals replicate the chronic course of the disease [13,34]. However, they are associated with animal welfare issues and limited reproducibility due to poorly controllable biochemical adaptations to developing dopaminergic deficits. Given that numerous variants of MPTP administration have been reported in the literature, there is a lack of clarity regarding which of the potential routes and dosing schemes are best suited for rapid screening of new PD-targeting therapies [35,36].

Bolus MPTP models are simple, inexpensive, and rapid in implementation; thus, this type of model can be valuable for the pre-clinical screening of new drugs [29]. A bolus administration of MPTP (40 mg/kg subcutaneously) was effective in inducing neurotoxicity and allowed for rapid evaluation of the potential effects of neuroprotective compounds in 9–10-month-old male C57BL/6J mice [29]. Acute repeated administration of high doses of MPTP, for example, of four intraperitoneal injections of 20 mg/kg within 24 h, caused the rapid death of dopaminergic neurons in 8-week-old male OF1 mice [35].

The goal of the present study was to address the effectiveness and validity of a simplified MPTP model based on a single intraperitoneal injection of 30 mg/kg MPTP to 12-month-old female mice that also allows better compliance with animal welfare in comparison with protocols using repeated or chronic toxin administration. In addition, we aimed to probe the sensitivity of MPTP-induced changes to the effects of known antioxidants fullerene C_60_ and fullerenol C_60_(OH)_2_ acting via peripheral or central mechanisms, respectively [11,34].

Fullerene C_60_ and fullerenol C_60_(OH)_24_ are compounds with antioxidant, anti-inflammatory, neuroprotective, and anti-aggregation properties [37,38,39,40,41,42]. Both compounds were shown to scavenge reactive oxygen and nitrogen species, including superoxide, hydroxyl radicals, and peroxynitrite [37]. In particular, Askarova et al. recently demonstrated the beneficial effects of chronic dosing with C_60_ and fullerenol C_60_(OH)_24_ on the hallmarks of Alzheimer‘s disorder in APPswe/PS1E9 mice [43]. Golomidova et al. demonstrated that fullerenols C_60_(OH)_30_ and C_70_(OH)_30_ exerted neuroprotective effects in a Drosophila melanogaster PD model, reducing alpha-synuclein aggregation and oxidative stress, normalizing dopamine levels, and dopaminergic neuron counts in the A53T mutants [41]. Accumulating evidence has shown the potential to counteract the neurodegeneration of fullerene C_60_ and fullerenol C_60_(OH)_24_ in models of Alzheimer’s disease, multiple sclerosis, PD, and others [38,43,44,45,46,47].

Fullerene C_60_ is a water-insoluble compound with limited bioavailability that has been shown to realize its activity through modulation of gut microbiome, as it does not penetrate the blood–brain barrier (BBB) [42,48], while fullerenol C_60_(OH)_24_ acts directly on the brain by crossing the BBB [48]. Fullerene C_60_ was found to accumulate in peripheral organs, mainly in the liver, which is not a characteristic of fullerenol C_60_(OH)_24_ that can be found in the brain and is rapidly eliminated through urine [48,49]. Interestingly, intragastric administration of fullerene C_60_ for 1–7 days at doses 1.1–6.5 mg/kg/day to young male C57BL/6 mice receiving MPTP for 5 consecutive days increased the richness and diversity of the gut microbiota in MPTP-injected mice and induced a distinct clustering of microbial community structure more closely resembling that of controls. Microbiota composition analysis revealed that C_60_-enriched populations of *Bacteroides*, *Akkermansia*, *Faecalibaculum*, and *Parabacteroides* are beneficial for gut structure and function. These microbial changes were accompanied by reduced microglial activation and decreased levels of brain pro-inflammatory cytokines, such as IL-1β and IL-6; enhanced intestinal barrier integrity; and ameliorated neuroinflammation and dopaminergic deficits in MPTP-injected mice [42]. It was proposed that these effects resulted in improved motor scores in the pole test and rotorod in MPTP-induced PD mice treated with C_60_. Unlike C_60_, C_60_(OH)_24_ exhibits good water solubility, rapidly crosses the BBB, and, as shown in the MPP^+^ cellular model of PD, preserves mitochondrial membrane potential; maintains the activity of respiratory chain complexes; reduces ROS, DNA, and protein oxidative damage; and restores glutathione levels and Nrf2-signaling [37,50,51,52].

Notably, C_60_(OH)_24_ was demonstrated to prevent amyloid fibril formation with the highest proficiency [39]. Both fullerene C_60_ and fullerenol C_60_(OH)_24_, administered at equimolar concentrations of 0.1 mg/kg/day and 0.15 mg/kg/day, respectively, to female APPswe/PS1E9 mice, a model of Alzheimer’s disorder, were recently shown to reduce disease manifestations [43]. Fullerene C_60_ and fullerenol C_60_(OH)_24_ exerted distinct ameliorative behavioral effects, where only the latter reduced amyloid burden and microglial activation and downregulated the pro-inflammatory cytokine interleukin (IL) *Il-1β* in the brain of APPswe/PS1E9 mutants [43].

Here, we tested the potential effects of 10-day dosing with fullerene C_60_ or fullerenol C_60_(OH)_24_ administered in equimolar concentrations of 0.1 mg/kg/day and 0.15 mg/kg/day in a bolus MPTP model in aged female C57BL/6J mice. Considering the short dosing period applied in the present study, we assessed the possibility of increasing the drug concentration by studying the tolerability of mice to three fullerenol concentrations in a separate study, as fullerenols may exert toxic effects [53,54]. The selection of the dose used was based on the outcome of this experiment, a recent study on APPswe/PS1E9 mice [43], and previous publications [43,44,45,55,56,57].

Following the determination of the doses of fullerene C_60_ and fullerenol C_60_(OH)_24_ at the three investigated doses, the medium concentration was selected and administered for ten days, followed by intraperitoneal injection of MPTP. Immediately thereafter, we scored commonly accepted manifestations of vegetative response to MPTP in mice: tremor and piloerection, as well as changes in motor parameters in the pole test, rotarod, open field, and walking path. Biochemical measures of dopamine metabolism, including levels of dopamine (DA) and its major metabolites 3,4-dihydroxyphenylacetic acid (DOPAC), homovanillic acid (HVA), and 3-methoxytyramine (3-MT), a marker of dopamine release into the extracellular space, were determined using high-performance liquid chromatography (HPLC) in the striatum and prefrontal cortex of experimental mice.

In our study, in addition to the striatum, we chose to address neurochemical and molecular MPTP-induced changes in the prefrontal cortex, given its role in behavioral non-motor symptoms of PD [2,58] and the strong anatomical and functional interconnection between these two brain structures [59,60]. The two brain structures were examined for PCR gene expression of markers of nitrosative stress: neuronal nitric oxide synthase (NOS) (nNOS), inducible NOS (iNOS), and endothelial NOS (eNOS), which mediate dopaminergic neurodegeneration in PD [61], and of important regulators of dopaminergic transmission driving oxidative stress and neuronal loss in PD, monoamine oxidase (MAO)-A and MAO-B [62,63], catechol-O-methyltransferase (COMT), which regulates dopamine degradation [64], and tyrosine hydroxylase (TH), which catalyzes the rate-limiting step in dopamine synthesis [65], and suppressed MPTP-treated mice, correlating with dopaminergic neuron loss [66].

The gene expression encoding alpha-synuclein (SNCA), which is upregulated in PD brain tissue and MPTP-treated mice, aggregates into Lewy bodies [67] and synaptophysin (*Syp*), a marker of synaptic density whose striatal expression is decreased following MPTP exposure [68]. Finally, we investigated the expression of the gene encoding peroxisome proliferator-activated receptor-gamma coactivator mitochondrial marker PGC-1α, whose expression is linked to nitrosative stress and has been shown to be reduced in PD patients [69].

## 2. Materials and Methods

### 2.1. Animals and Housing

Female C57BL/6J mice, aged 12 months, were obtained from a licensed commercial supplier (the Centre for Collective Use IPAC RAS) and housed under standard laboratory conditions (12 h light/12 h dark cycle; lights on at 21:00 h) with free access to diet and water. Mice were housed 5 per cage under controlled laboratory conditions. Temperature and humidity were maintained at 21–23 °C and 40–60%, respectively. The mice were acclimatized for one week prior to further procedures; all potential confounding factors were controlled. Observations of the animals were conducted each morning and evening throughout the experimental period. All procedures were approved by the Institutional Animal Care and Use Committee of the Institute of Molecular Biology of NAS RA (IMB1212-24, date of approval: 12 December 2024) and conducted in accordance with the European Directive 2010/63/EU on the protection of animals used for scientific purposes. The study did not include humane endpoints.

### 2.2. Experimental Design

**Figure 1:**
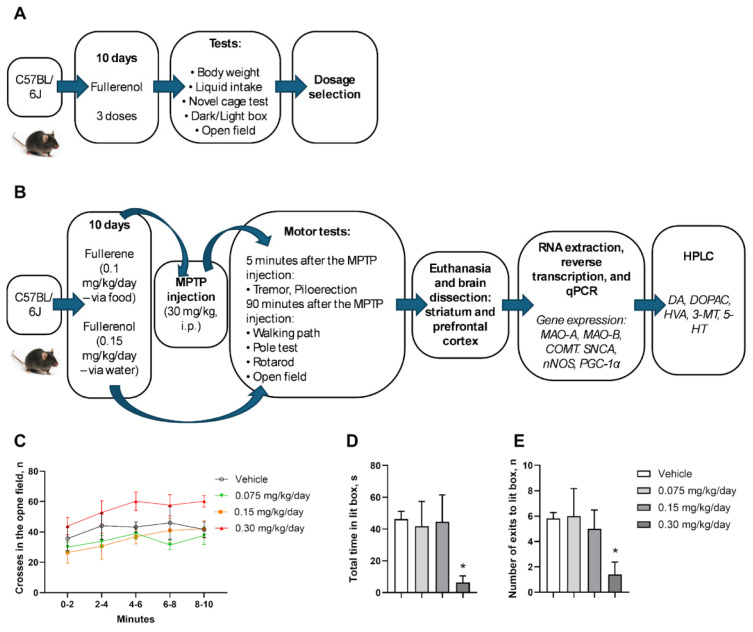
**Experimental design and results of the study with C_60_(OH)_24_ dose definition:** (**A**) Study flow of the first experiment. (**B**) Study flow of the second experiment with MPTP injection. No significant group differences were found in (**C**) weight gain, (**D**) Total time spent in the light compartment of the dark-light box and (**E**) total number of exits were significantly lower in the group treated with C_60_(OH)_24_ at the dose of 0.30 mg/kg/day compared with the non-treated group. * *p* < 0.05.

Two studies were carried out. In the first study, mice received C_60_(OH)_24_ dissolved in a tap water in either low (0.075 mg/kg/day; *n* = 5), medium (0.15 mg/kg/day; *n* = 5) or high (0.30 mg/kg/day; *n* = 5) concentration, or tap water (n = 5), for ten consecutive days. In total, 20 mice were used; randomization was done by body weight prior to the onset of treatment. Liquid and diet intake, as well as body weight, were monitored as described elsewhere [70]. By the end of the dosing, all mice were studied in the open field and dark-light box tests (Figure 1A).

In the second study, mice were allocated to one of the following groups: vehicle control (saline; *n* = 5), control C_60_-treated (*n* = 5), control C_60_(OH)_24_-treated (*n* = 5), MPTP only (*n* = 10), MPTP + C_60_ (*n* = 10), and MPTP+ C_60_(OH)_24_ (*n* = 10); randomization was done by body weight prior the onset of treatment. C_60_ (0.1 mg/kg/day) and C_60_(OH)_24_ (0.15 mg/kg/day) were administered for 10 consecutive days, as described elsewhere [70]. On day 10, mice received a single intraperitoneal injection of MPTP (30 mg/kg, freshly prepared in 0.9% NaCl) or vehicle, that were administered between 09:00 and 11:00 h. Immediately thereafter, mice were studied for tremor and piloerection. 90 min following MPTP administration, the step length of all mice was measured, and thereafter all animals were consequently investigated in the pole test, rotarod, and the open field. 12 h post-injection, all mice were killed, and their brains were dissected for the striatum and prefrontal cortex, which were used for subsequent HPLC and RT-PCR assays (Figure 1B). In this experiment, 45 mice were used. In total, 65 mice were used in the study. No criteria were set for including and excluding animals. Experimenters were blinded to groups until data analysis. The sample size was determined based on prior studies employing this model [16,36] and treatment with fullerene and fullerenol, as described elsewhere [43].

### 2.3. Drug Administration and Evaluation of Diet and Liquid Intake

Fullerene C_60_ (0.1 mg/kg/day) was administered via self-prepared food pellets, since it is non-soluble. Food pellets were prepared as described elsewhere [70]. In both studies, diet intake and drinking behavior were monitored during the dosing period [71,72]. In the first study, fullerenol C_60_(OH)_24_ (0.075, 0.15, or 0.30 mg/kg/day) was administered via tap water, with solutions replaced every 3 days. No group differences in fluid intake were observed, suggesting normal drinking behavior. The choice of doses for the second study was based on previous studies demonstrating effectiveness in neurodegeneration models [43] and results of behavioral assessment in the first experiment. In a recent study, fullerenol showed no cytotoxicity in CTX TNA2 astrocytes or bEnd.3 endothelial cells, and low concentrations (0.1–1 µg/mL) had no significant effect on BV2 microglial cell viability, confirming its low toxicity in these neural cell types [43]. Thus, based on this and other in vitro [73] and in vivo [53] studies, the concentrations of fullerene and fullerenol utilized here appear to be well below the toxic levels. Body weight was monitored daily to assess the general physical state of experimental animals in both studies.

### 2.4. Assessment of Vegetative Parkinsonian-like Response to MPTP

Following MPTP administration, tremor and piloerection were assessed during a 5 min observation period, as described elsewhere [74]. The latency to tremor onset was measured, and tremor intensity was scored on a semi-quantitative scale (1–5) by two independent observers. In brief, tremor intensity was scored on a 0–5 scale: 0, no tremor; 1, occasional muscle twitches or a barely visible tremor localized to the head; 2, moderate, intermittent tremor limited to the head region; 3, clear tremor with occasional quiescent periods, affecting the anterior part of the body; 4, continuous tremor involving the head and extremities; 5, continuous, pronounced, whole-body tremor, as previously described [75]. The percentage of mice with high tremor scores (4–5) was calculated, and the presence or absence of piloerection was recorded [36]. Control groups were excluded from this analysis.

### 2.5. Motor and Behavioural Tests

#### 2.5.1. Walking Path Assessment

All mice, including the control groups, underwent walking path assessment as described by Wertman et al. [76]. Non-toxic, washable tempera paint (two contrasting colors, green and purple) was applied to forelimbs and hind limbs. Mice were allowed to walk a narrow path (8 × 40 cm) over paper, producing footprint trails. Immediately after that, mice were retrieved and returned to their home cage with their feet wiped with a water-dampened cloth. Paper with footprints was allowed to dry fully before scoring. The testing area was wiped down with a cleaning solution in between each animal.

#### 2.5.2. Pole Test

The pole test was performed 90 min after MPTP injection as described elsewhere [77] to assess motor coordination and bradykinesia. Mice were placed on top of the vertical bar (diameter 1.1 cm, height 60 cm) and allowed to climb down to a horizontal surface. Latency to turn and latency to descend were recorded for each animal from the time they were placed head-up at the top of the pole. The percentage of mice that were unable to descend in 2 min was calculated for the MPTP-injected groups.

#### 2.5.3. Rotarod

Mice were placed on a constantly rotating rod of rotarod (Columbus Instruments, Columbus, OH, USA) for 5 min (speed 10 rpm). Latency to fall was registered as described elsewhere [77]. All mice were pre-trained on day 9 to familiarize them with the apparatus and reduce novelty-related variability during the post-MPTP administration period.

#### 2.5.4. Open Field

The test was conducted in a square open-field arena (45 × 45 × 45 cm; Technosmart, Rome, Italy) illuminated with dim light (5 lux). Each mouse was placed near one of the walls, and its behavior was recorded using the EthoVision XT software (version 6.95; Noldus, Wageningen, The Netherlands). In the first experiment, locomotor activity was tracked for 10 min, and the number of crossed sectors was analyzed in 2-min intervals. In the second experiment, the observation period lasted 5 min, and the number of crossed sectors was recorded for peripheral and central (15 × 15 cm) parts and the number of freezing episodes of the arena for the 1-min intervals, as previously described [78].

#### 2.5.5. Dark–Light Box

The apparatus (Technosmart, Rome, Italy) consisted of dark and illuminated (5 lx) compartments (both 20 × 20 × 25 cm). The mice were introduced into the dark compartment and allowed to move freely between the two chambers. The total number of exits to the lit compartment and the total time spent in the compartment were scored for 5 min, as described elsewhere [79].

### 2.6. Killing of Mice and Tissue Collection

Mice were terminally anesthetized by isoflurane inhalation and then perfused with ice-cold saline via the left ventricle; halves of the brains were removed, as described elsewhere [80]. Their striatum and prefrontal cortex were dissected, snap-frozen in dry ice, and stored at −80 °C until use.

### 2.7. High-Performance Liquid Chromatography (HPLC) Assay

To assess dopaminergic neurotransmission, HPLC with electrochemical detection was performed on striatal and cortical samples. Concentrations of striatal dopamine (DA), 3,4-dihydroxyphenylacetic acid (DOPAC), and homovanillic acid (HVA) in extracts obtained by homogenization in 0.06 M HClO^4^ (Sigma Aldrich, Saint Louis, MO, USA) followed by centrifugation at 15,000× *g* for 5 min at 4 °C were measured by HPLC as described previously [81]. According to the manufacturer’s instructions, the protein concentrations in the samples afterward were measured using a Pierce™ BCA Protein Assay kit (Thermo Scientific, Waltham, MA, USA).

### 2.8. RNA Extraction, cDNA Synthesis, and Real-Time Polymerase Chain Reaction

Total RNA of the prefrontal cortex and striatum tissue samples was isolated using QIAzol^®^ Lysis Reagent (QIAGEN Sciences Inc., Germantown, MD, USA). Tissue was placed in 1 mL of QIAzol, homogenized using a TissueRuptor (QIAGEN Sciences Inc., Germantown, MD, USA) with two 30 s cycles of the homogenizer at half speed, and then placed on ice for one minute after every homogenization. Homogenized samples were centrifuged for 15 min at 12,000× *g* and 4 °C to remove any remaining cell debris. Chloroform was then added to the homogenized sample and centrifuged for 15 min at 12,000× *g* at 4 °C, and the aqueous phase was carefully removed. RNA precipitation was performed with the addition of ethanol, and the subsequent RNA pellet was washed and cleaned using the RNeasy Mini Kit (QIAGEN Sciences Inc., Germantown, MD, USA). The RNA concentration was measured using a NanoDrop spectrophotometer (Thermo Fisher Scientific, Waltham, MA, USA).

The total RNA (1 μg) was converted to cDNA. First-strand cDNA synthesis was performed using random primers and a QuantiTect Reverse Transcription Kit (QIAGEN Sciences Inc., Germantown, MD, USA). The reaction was performed in an Eppendorf Mastercycler^®^ Gradient using the following temperature program: 68 °C for 5 min, 42 °C for 60 min, and 70 °C for 10 min. To identify the expression levels of the target genes, we designed gene-specific primers. The housekeeping glyceraldehyde 3-phosphate dehydrogenase (*Gapdh*) gene was used as a reference. The sequences of the designed primers are listed in Table S2 (see Supplementary File). The expression levels of genes of interest were determined using real-time polymerase chain reaction (RT-PCR). RT-PCR was performed using the SYBR Green Master Mix (Applied Biosystems SYBR Green Universal Master Mix, Foster City, CA, USA). RT-PCR was performed in a 10 μL reaction volume containing SYBR Green master mix (5 µL), RNase-free water (3 μL), specific forward and reverse primers used at a concentration of 20 pmol/µL (1 μL), and cDNA (1 μL). The initial denaturation step for RT-PCR was performed at 95 °C for 5 min, followed by 40 cycles of denaturation at 95 °C for 20 s, annealing at 60 °C for 30 s, and extension at 68 °C for 30 s. All primers were purchased from Sigma-Aldrich (USA). All samples were run in triplicate. Reactions were performed using an ABI Prism 7900 HT SDS instrument (Applied Biosystems, Foster City, CA, USA). Data were normalized to *Gapdh* mRNA expression and calculated as relative-fold changes compared to the control vehicle group, as described elsewhere [80].

### 2.9. Statistical Analysis

Data were analyzed using GraphPad Prism (version 8.0.2, San Diego, CA, USA). Normality was assessed with the Shapiro–Wilk test. For comparisons among three groups, one-way analysis of variance (ANOVA) followed by Tukey’s post hoc test was used, which enables adjustment of necessary corrections for multiple comparisons to avoid false-positive results. If the data did not meet normality assumptions, the Kruskal–Wallis test was applied instead. For comparisons among six groups, a two-way ANOVA was performed. Categorical outcomes were analyzed using Fisher’s exact test for contingency tables. Cohen’s d was calculated for all statistically significant paired comparisons as a measure of effect size that quantifies the magnitude of the difference between two group means. No data points were excluded from the analysis. Statistical significance was set at *p* < 0.05. Data are presented as mean ± SEM, with group sizes specified in the Materials and Methods section and in Figure legends.

## 3. Results

### 3.1. Effects of Different C_60_(OH)_24_ Doses on Behavioral Outcomes in Naïve Mice

In the first experiment, no significant group differences were revealed for weight gain (*p* = 0.217, Kruskal–Wallis test; Figure 1C), as well as water consumption (F = 0.643, *p* = 0.598, one-way ANOVA; Figure 1D) or diet consumption (F = 0.718, *p* = 0.555, one-way ANOVA; Figure 1E).

**Figure 2:**
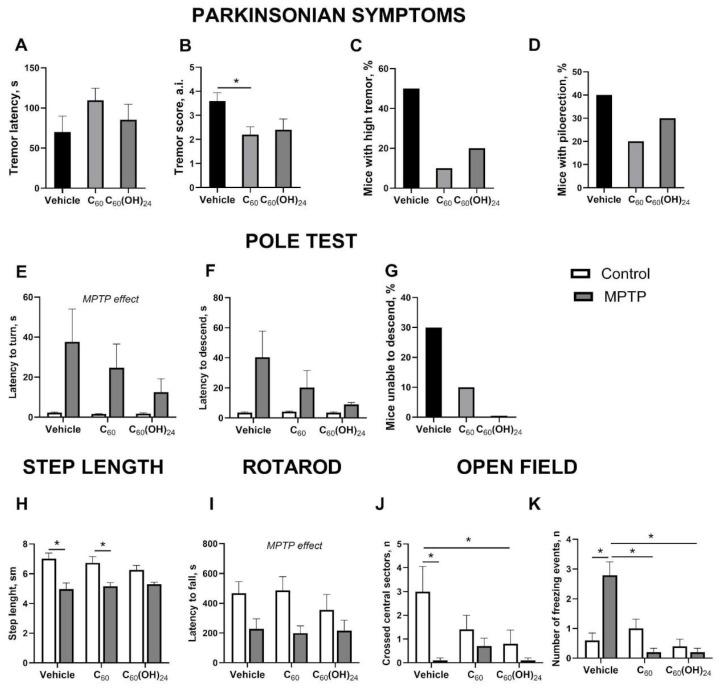
**Behavioral changes and vegetative outcomes following MPTP injection and dosing with C_60_ or C_60_(OH)_24_:** (**A**) Tremor score was significantly lower in MPTP-affected mice treated with C_60_ in comparison with the untreated mice. (**B**) No significant differences were found in tremor latency. (**C**) The percentage of mice with high tremor was significantly lower in MPTP-affected mice treated with C_60_ in comparison with the untreated mice. (**D**) No significant group differences were found in the percentage of mice with piloerection. (**E**) Step length was significantly shorter in the MPTP-affected non-treated group and the C_60_-treated group compared with the respective controls. In the pole test, no significant differences were revealed in (**F**) latency to turn, (**G**) latency to descend, and (**H**) percentage of mice unable to descend. (**I**) No significant group differences were found in latency to fall from the rotarod. (**J**) The number of crossed sectors in the open field was significantly lower in non-treated MPTP-affected mice and the C_60_(OH)_24_-treated control group compared to control non-treated mice. (**K**) The number of freezing events was significantly higher in the MPTP-affected non-treated group than in the control non-treated group, MPTP-affected C_60_- and C_60_(OH)_24_-treated mice. * *p* < 0.05, Fisher’s exact test, one-way or two-way ANOVA with post hoc Tukey’s test. The following groups were examined: vehicle control (saline; n = 5), control C_60_-treated (n = 5), control C_60_(OH)_24_-treated (n = 5), MPTP only (n = 10), MPTP + C60 (n = 10), and MPTP+ C_60_(OH)_24_ (n = 10). MPTP—1-methyl-4-phenyl-1,2,3,6-tetrahydropyridine, a.i.—arbitrary units. Data are presented as mean ± SEM.

Significant group differences were shown for total time spent in the light compartment of the dark–light box (F = 3.190, *p* = 0.0477, one-way ANOVA) and total number of exits (F = 3.104, *p* = 0.0474). Both these measures were significantly lower only in the group treated with C_60_(OH)_24_ at the dose of 0.30 mg/kg/day compared with the non-treated group (*p* = 0.0378 and *p* = 0.0422, respectively, Tukey’s test; Figure 1F,G). In the open field, significant group differences were found in the total number of crossed sectors (F = 11.16, *p* < 0.0001, two-way ANOVA). Specifically, mice treated with C_60_(OH)_24_ at the dose of 0.30 mg/kg/day crossed significantly more sectors during minutes 6–8 than animals treated with C_60_(OH)_24_ at the dose of 0.075 mg/kg/day (*p* = 0.0231, Tukey’s test; Figure 2H).

### 3.2. Motor Functions and Vegetative Outcomes in Mice Subjected to Dosing with C_60_ or C_60_(OH)_24_ and MPTP Injection

One-way ANOVA revealed significant group differences in tremor score (F = 4.031, *p* = 0.0294). Specifically, C_60_ significantly decreased tremor score in MPTP-affected mice in comparison with the non-treated group (*p* = 0.0364, post hoc Tukey’s test; Figure 2A), with a similar trend shown for C_60_(OH)_24_ (*p* = 0.0808). At the same time, latency to tremor did not differ significantly between groups (F = 1.231, *p* = 0.308; Figure 2B). The percentage of mice that demonstrated high tremor scores (4 or 5) was significantly lower in the C_60_-treated but not in the C_60_(OH)_24_-treated group (*p* = 0.0704 and *p* = 0.174, respectively, Fisher’s exact test; Figure 2C). No significant group differences were found in the percentage of mice that showed piloerection following MPTP injection (*p* > 0.05, Fisher’s exact test; Figure 2D). Significant MPTP effect but not treatment or their interaction was observed for step length in the walking path (F = 41.65, *p* < 0.0001, F = 0.246, *p* = 0.782, and F = 1.31, *p* = 0.279, respectively, two-way ANOVA). Specifically, it was significantly shorter in the MPTP-injected non-treated group (*p* = 0.0012, Tukey’s test) and the C_60_-treated group (0.027) but not in the C_60_(OH)_24_-treated group (*p* = 0.24; Figure 2E) compared with respective controls.

Significant MPTP effect was shown for latency to turn in the pole test (F = 5.09, *p* = 0.0297, two-way ANOVA; Figure 2F), but no effects of treatment or their interaction were found (F = 0.513, *p* = 0.604 and F = 0.481, *p* = 0.621, respectively). Similarly, a strong trend for MPTP effect was shown for latency to descend (F = 3.802, *p* = 0.0584, two-way ANOVA; Figure 2G), but not for treatment or interaction (F = 0.843, *p* = 0.438 and F = 0.855, *p* = 0.433, respectively). Post hoc analysis did not show any significant differences for these parameters (*p* > 0.05, Tukey’s test). No significant differences were revealed in the percentage of mice that were unable to descend from the pole between MPTP-affected groups (*p* > 0.05, Fisher’s exact test; Figure 2H). Next, significant MPTP effect was revealed also for latency to fall from the rotarod (F = 15.02, *p* = 0.0003, two-way ANOVA; Figure 2I), without significant effects of treatment and interaction (F = 0.56, *p* = 0.643 and F = 0.3, *p* = 0.825, respectively); post hoc analysis did not reveal any significant differences (*p* > 0.05, Tukey’s test).

Significant effects of MPTP, treatment, and their interaction were revealed for the number of crossed central sectors during the first minute of the open field (F = 16.72, *p* = 0.0002; F = 3.293, *p* = 0.0477, and F = 4.379, *p* = 0.0193, respectively; two-way ANOVA). This measure was significantly lower in non-treated MPTP-injected mice as well as C_60_(OH)_24_-treated control group compared to control non-treated mice (*p* = 0.0003 and *p* = 0.0354, respectively, Tukey’s test; Figure 2J). No significant effects of MPTP were shown for the number of freezing events (F = 2.476, *p* = 0.123, two-way ANOVA), though treatment and MPTP × treatment interaction significantly affected this parameter (F = 11.21, *p* = 0.0001, and F = 13, *p* < 0.0001, respectively). The number of freezing events was significantly higher in the MPTP-challenged non-treated group than in the control non-treated group (*p* = 0.0002, Tukey’s test) and both MPTP-injected C_60_- and C_60_(OH)_24_-treated mice (both *p* < 0.0001; Figure 2K). No other changes were found to be significant in the open-field test.

### 3.3. MPTP-Induced Neurochemical Changes in the Striatum and Effects of Dosing with C_60_ or C_60_(OH)_24_

**Figure 3:**
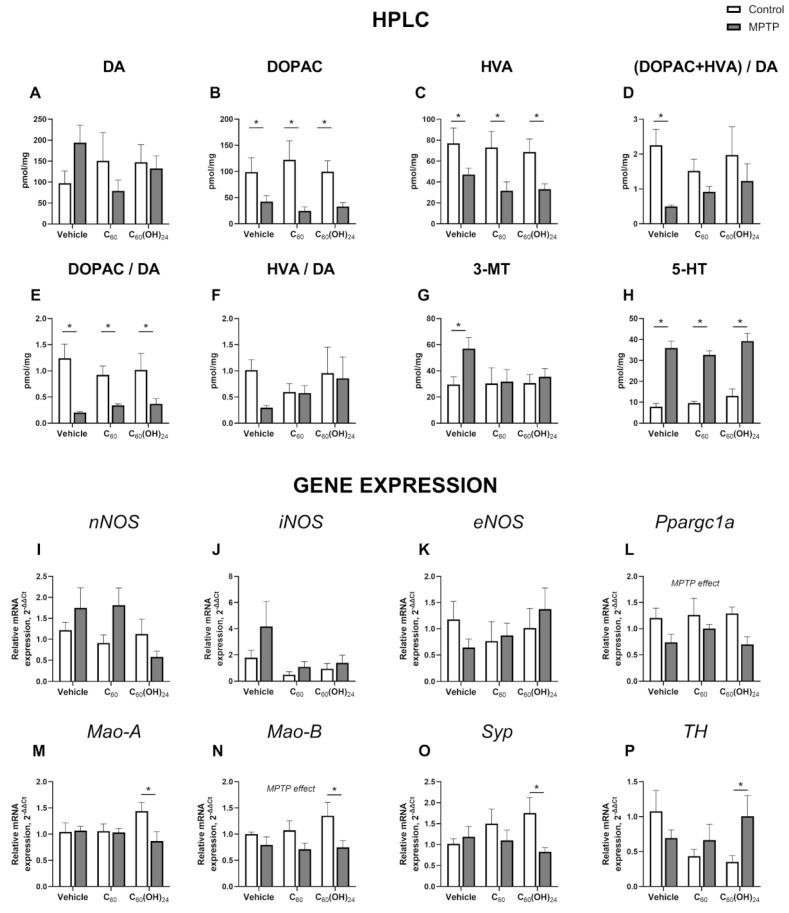
**Molecular changes in the striatum following MPTP injection and dosing with C_60_ or C_60_(OH)_24_**: (**A**) Overall dopamine (DA) levels in the striatum were not significantly affected by MPTP treatment or the compounds. (**B**) Levels of dopamine metabolite 3,4-dihydroxyphenylacetic acid (DOPAC) in the striatum were significantly decreased due to MPTP treatment. (**C**) Levels of dopamine metabolite homovanillic acid (HVA) in the striatum were significantly decreased due to MPTP treatment. (**D**) The sum of DOPAC and HVA divided by dopamine was significantly decreased in the striatum of the MPTP-affected group, while being ameliorated by both C_60_ and C_60_(OH)_24_. (**E**) MPTP significantly decreased the level of DOPAC divided by DA in the striatum. (**F**) There were no significant changes in the levels of HVA divided by DA in the striatum. (**G**) Levels of 3-methoxytyramine (3-MT) in the striatum were significantly increased in the MPTP-treated group, while being ameliorated by both C_60_ and C_60_(OH)_24_. (**H**) Levels of serotonin (5-HT) in the striatum were significantly increased by MPTP. Neither MPTP nor the compounds C_60_ and C_60_(OH)_24_ affected the expression of NO-synthases (**I**) *nNOS*, (**J**) *iNOS*, and (**K**) *eNOS*. (**L**) *Ppargc1α* expression was not affected in the striatum. (**M**) C_60_(OH)_24_ significantly decreased *Mao-A* expression in the MPTP-affected group in comparison with the control. (**N**) *Mao-B* expression was significantly decreased in the MPTP-affected groups, and C_60_(OH)_24_ showed a trend to increase *Mao-B* expression in the striatum. (**O**) There was a significant difference in *Syp* expression between the MPTP-affected and non-affected C_60_(OH)_24_-treated groups in the striatum. (**P**) There was a significant difference in *TH* expression between the MPTP-affected and non-affected C_60_(OH)_24_-treated groups in the striatum. * *p* < 0.05, two-way ANOVA with post hoc Tukey’s test. The following groups were examined: vehicle control (saline; *n* = 5), control C_60_-treated (*n* = 5), control C_60_(OH)_24_-treated (*n* = 5), MPTP only (*n* = 10), MPTP + C60 (*n* = 10), and MPTP+ C_60_(OH)_24_ (*n* = 10). Data are presented as mean ± SEM.

No significant effects of MPTP, treatment, or their interaction were revealed for the overall dopamine levels in the striatum (F = 0.01040, *p* = 0.9194; F = 0.3036, *p* = 0.7402, and F = 2.239, *p* = 0.1225, respectively; two-way ANOVA; Figure 3A). Significant effect of MPTP but not treatment or interaction was observed for the overall levels of the dopamine metabolite 3,4-dihydroxyphenylacetic acid (DOPAC) in the striatum (F = 30.70, *p* < 0.0001; F = 0.1064, *p* = 0.8994 and F = 0.7569, *p* = 0.4771, respectively; two-way ANOVA; Figure 3B), with significant changes observed for the non-treated group and the groups treated with C_60_ or C_60_(OH)_24_ (*p* = 0.0175, *p* = 0.0007 and *p* = 0.0021, respectively; Tukey’s test). Similarly, levels of homovanillic acid (HVA) were significantly affected by MPTP administration (F = 19.67, *p* < 0.0001; two-way ANOVA; Figure 3C) and not treatment or interaction (F = 0.8273, *p* = 0.4461 and F = 0.1512, *p* = 0.8602, respectively), with significant reductions observed in the same three groups (*p* = 0.0356, *p* = 0.0132 and *p* = 0.0057, respectively; Tukey’s test).

The combined ratio of DOPAC and HVA to dopamine was significantly influenced by MPTP but not treatment or interaction (F = 6.182, *p* = 0.0181; F = 0.2975, *p* = 0.7446 and F = 0.7662, *p* = 0.4729, respectively; two-way ANOVA; Figure 3D), with significant alterations in the vehicle group (*p* = 0.0183), which were ameliorated by both C_60_ and C_60_(OH)_24_ (*p* = 0.4668 and *p* = 0.2403, respectively). DOPAC/dopamine ratios were also significantly impacted by MPTP and not treatment or interaction (F = 28.29, *p* < 0.0001; F = 0.1234, *p* = 0.8843 and F = 1.000, *p* = 0.3786, respectively; two-way ANOVA; Figure 3E), with changes found in all groups (*p* = 0.0001, *p* = 0.0448 and *p* = 0.0046, respectively; Tukey’s test). However, no significant changes were found in HVA-to-dopamine ratios (F = 0.8958, *p* = 0.3508; F = 0.4842, *p* = 0.6205 and F = 0.5665, *p* = 0.5729, respectively; two-way ANOVA; Figure 3F).

For 3-methoxytyramine (3-MT), no significant effects of MPTP, treatment or their interaction were revealed (F = 2.514, *p* = 0.1224; F = 1.142, *p* = 0.3316 and F = 1.356, *p* = 0.2717, respectively; two-way ANOVA; Figure 3G); however, Tukey’s test revealed that 3-MT levels were significantly increased in the MPTP-affected control group (*p* = 0.0286), while being ameliorated in both the C_60_- and C_60_(OH)_24_-treated groups (*p* = 0.9202 and *p* = 0.6592, respectively). Significant effect of MPTP but not treatment or interaction was observed for serotonin (5-HT) in the striatum (F = 83.45, *p* < 0.0001; F = 1.377, *p* = 0.2665 and F = 0.2359, *p* = 0.7912, respectively; two-way ANOVA; Figure 3H), with significant changes observed for the non-treated group and the groups treated with C_60_ or C_60_(OH)_24_ (*p* < 0.0001, *p* = 0.0002 and *p* < 0.0001, respectively; Tukey’s test).

There were no significant effects of MPTP, treatment, or their interaction revealed for the expression of *nNOS* (F = 0.9808, *p* = 0.3290; F = 1.712, *p* = 0.1958 and F = 2.233, *p* = 0.1227, respectively; two-way ANOVA; Figure 3I), *iNOS* (F = 1.719, *p* = 0.1998; F = 2.320, *p* = 0.1156 and F = 0.4876, *p* = 0.6189, respectively; Figure 3J), or *eNOS* (F = 0.006349, *p* = 0.9370; F = 0.5820, *p* = 0.5652 and F = 0.9520, *p* = 0.3977, respectively; Figure 3K). We found no significant effects of MPTP on *Ppargc1α* expression, while no significant effects of treatment or their interaction were shown for this molecule (F = 1.118, *p* = 0.3018; F = 0.5369, *p* = 0.5920, and F = 0.5461, *p* = 0.5868, respectively; Figure 3L).

ANOVA showed no significant effects of MPTP, treatment or their interaction were revealed for *Mao-A* expression (F = 2.947, *p* = 0.1001; F = 0.3819, *p* = 0.6870 and F = 2.901, *p* = 0.0762, respectively; Figure 3M), although Tukey’s test revealed a significant difference in the C_60_(OH)_24_-treated group (*p* = 0.0079). *Mao-B* expression was significantly affected by MPTP but not treatment or their interaction (F = 10.14, *p* = 0.0047; F = 0.6754, *p* = 0.5202, and F = 0.8619, *p* = 0.4374, respectively; Figure 3N), with significant differences in the C_60_(OH)_24_-treated group (*p* = 0.0139).

No significant effects of MPTP, treatment, or interaction were revealed for *Syp* expression (F = 3.533, *p* = 0.0735; F = 0.4016, *p* = 0.6740, and F = 2.393, *p* = 0.1147, respectively; Figure 3O), though Tukey’s test indicated a significant difference in the C_60_(OH)_24_-treated group (*p* = 0.0176). Similarly, *TH* expression was not significantly affected by MPTP, treatment, or interaction (F = 0.9394, *p* = 0.3453; F = 1.270, *p* = 0.3049, and F = 3.098, *p* = 0.0698, respectively; Figure 3P), though Tukey’s test showed a significant effect in the C_60_(OH)_24_-treated group (*p* = 0.0374).

**Figure 4:**
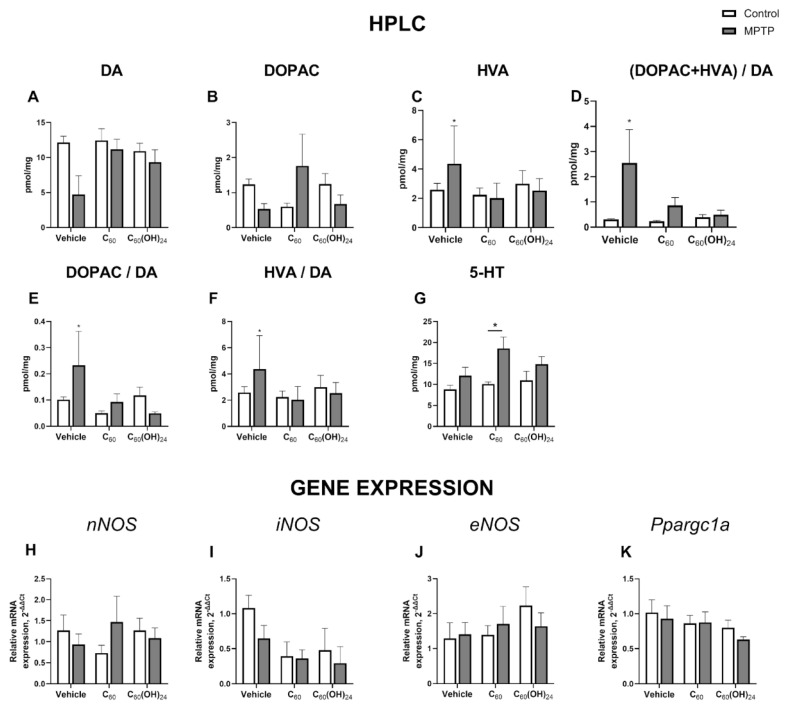
**Molecular changes in the prefrontal cortex following MPTP injection and dosing with C_60_ or C_60_(OH)_24_:** (**A**) Overall dopamine (DA) levels in the prefrontal cortex were not significantly affected by MPTP treatment or the compounds. (**B**) Levels of dopamine metabolite 3,4-dihydroxyphenylacetic acid (DOPAC) in the prefrontal cortex were not significantly affected by MPTP treatment or the compounds. (**C**) Levels of dopamine metabolite homovanillic acid (HVA) in the prefrontal cortex were not significantly affected by MPTP treatment or the compounds. (**D**) The sum of DOPAC and HVA divided by dopamine in the prefrontal cortex was not significantly affected by MPTP treatment or the compounds. (**E**) There were no significant changes in the levels of DOPAC divided by DA in the prefrontal cortex. (**F**) There were no significant changes in the levels of HVA divided by DA in the prefrontal cortex. (**G**) Levels of serotonin (5-HT) in the prefrontal cortex were significantly increased in the MPTP-affected C_60_-treated group. Neither MPTP nor the compounds C_60_ and C_60_(OH)_24_ affected the expression of NO-synthases (**H**) *nNOS*, (**I**) *iNOS*, and (**J**) *eNOS*, as well as (**K**) *Ppargc1α*.* *p* < 0.05, two-way ANOVA with post hoc Tukey’s test. The following groups were examined: vehicle control (saline; *n* = 5), control C_60_-treated (*n* = 5), control C_60_(OH)_24_-treated (*n* = 5), MPTP only (*n* = 10), MPTP + C60 (*n* = 10), and MPTP + C_60_(OH)_24_ (*n* = 10). Data are presented as mean ± SEM.

In the prefrontal cortex, no significant effects for MPTP, treatment or interaction were observed in overall dopamine levels (F = 2.098, *p* = 0.1569; F = 0.5082, *p* = 0.6062; F = 0.4175, *p* = 0.6621; two-way ANOVA; Figure 4A), DOPAC (F = 0.005949, *p* = 0.9390; F = 0.1248, *p* = 0.8831; F = 1.459, *p* = 0.2470; Figure 4B), or HVA (F = 0.2585, *p* = 0.6145; F = 0.5811, *p* = 0.5649; F = 0.9761, *p* = 0.3874; Figure 4C). MPTP, treatment, and interaction effects had no significant effect on the DOPAC + HVA/DA ratio (F = 1.445, *p* = 0.2388; F = 0.1440, *p* = 0.8665; F = 0.8876, *p* = 0.4222; Figure 4D), DOPAC/DA (F = 0.4966, *p* = 0.4866; F = 1.369, *p* = 0.2703; F = 1.365, *p* = 0.2712; Figure 4E), and HVA/DA (F = 1.704, *p* = 0.2013; F = 0.8806, *p* = 0.4247; F = 0.9694, *p* = 0.3905; Figure 4F), while the DOPAC + HVA/DA ratio and DOPAC/DA were increased in vehicle-treated MPTP-challenged groups but not in dosed animals. Serotonin levels were significantly influenced by MPTP and not treatment or interaction (F = 7.073, *p* = 0.0120; F = 1.241, *p* = 0.3023, and F = 0.6729, *p* = 0.5171, two-way ANOVA; Figure 4G) with significant changes observed in the C_60_-treated group (*p* = 0.0195, Tukey’s test).

We found no significant effects of MPTP, treatment or their interaction in the prefrontal cortex for the expression of *nNOS* (F = 0.05287, *p* = 0.8196; F = 0.02512, *p* = 0.9752; F = 1.007, *p* = 0.3766; Figure 4H), *iNOS* (F = 1.435, *p* = 0.2400; F = 2.998, *p* = 0.0645; F = 0.4033, *p* = 0.6716; Figure 4I), *eNOS* (F = 0.01851, *p* = 0.8927; F = 0.8714, *p* = 0.4290; F = 0.6039, *p* = 0.5534; Figure 4J), and *Ppargc1α* (F = 0.5129, *p* = 0.4826; F = 1.872, *p* = 0.1811; F = 0.2483, *p* = 0.7826; Figure 4K).

### 3.4. A Sensitivity Statistical Analysis of Effect Sizes

We conducted a sensitivity analysis tailored to our 2 × 3 design with the actual sample sizes (control group, n = 5 per group; MPTP groups, *n* = 10 per group; total, *n* = 45). Effect sizes were expressed as partial η^2^, which is the proportion of variance explained by a factor after controlling for other sources of variance. Partial η^2^ is standard in ANOVA and provides an interpretable scale, where values of ~0.01, ~0.06, and ~0.14 correspond to small, medium, and large effects, respectively. Using the relationship between η^2^ and Cohen’s f (η^2^ = f^2^/(1 + f^2^)), we determined the minimum detectable effect sizes. This analysis shows that with α = 0.05 and 80% power, our design could reliably detect large effects, approximately η^2^ ≥ 0.15 for the MPTP main effect, η^2^ ≥ 0.19 for the treatment main effect, and η^2^ ≥ 0.25 for the MPTP × treatment interaction. In practice, this means that medium or smaller effects may not reach significance, given the present sample sizes. For the one-way ANOVA used to determine the effective fullerenol dose (*n* = 5 per group; total *n* = 20), a similar sensitivity analysis indicated 80% power to detect large effects of approximately η^2^ ≥ 0.33 (Cohen’s f ≈ 0.71).

The analysis shows that with α = 0.05 and 80% power, our design could reliably detect large effects, approximately η^2^ ≥ 0.15 for the MPTP main effect, η^2^ ≥ 0.19 for the treatment main effect, and η^2^ ≥ 0.25 for the MPTP × treatment interaction. In practice, this means that medium or smaller effects may not reach significance given the present sample sizes. For the one-way ANOVA used to determine the effective fullerenol dose (*n* = 5 per group; total *n* = 20), a similar sensitivity analysis indicated 80% power to detect large effects of approximately η^2^ ≥ 0.33 (Cohen’s f ≈ 0.71).

## 4. Discussion

Aged female mice subjected to a bolus MPTP intraperitoneal injection at a dose of 30/mg/kg manifested Parkinsonian-like vegetative symptoms, disrupted motor functions, elevated anxiety, and profoundly impaired dopaminergic neurotransmission in the striatum and prefrontal cortex. These changes are accompanied by altered gene expression of molecular markers of nitrosative stress and several factors involved in dopaminergic regulation in these brain structures. Subchronic administration of either insoluble fullerene or water-soluble C_60_(OH)_24_ to the experimental groups of mice exerted limited but significant effects on several physiological and neurochemical MPTP-induced abnormalities. Thus, the present study demonstrates the good face validity of the MPTP model variant of the PD paradigm in aged female mice and suggests its usefulness for the efficient screening of drug candidates with antioxidant properties acting either via central or peripheral mechanisms.

Specifically, we found a significant reduction in tremor scores in C_60_-treated mice. Compared to the vehicle-treated MPTP-exposed group, the percentage of mice displaying intense tremor (score ≥ 4) was significantly smaller in C_60_-treated mice. These data suggest beneficial effects of fullerene on the Parkinsonian vegetative response to MPTP administration in this mode. Motor tests revealed robust effects of MPTP on the parameters of descent and turning in the pole test, rotarod, and step path assay. Our study revealed a strong increase in the latency to descent and the latency to turn in the pole test following MPTP administration, which manifested PD-like motor rigidity, one of the key symptoms of PD. Among fullerenol-treated MPTP mice, this measure was significantly lower in the C_60_(OH)_24_-treated group than in the vehicle-treated MPTP animals. Similarly, we observed a strong reduction in path length, which is a sign of severe motor dysfunction in MPTP-challenged mice. In the rotarod test, the latency to fall decreased in all MPTP groups regardless of the treatment. Together, the present data show modest but significant normalizing effects of fullerenol on the motor behavior of MPTP-challenged mice that were not found for the fullerene-treated group.

In the open-field test, we found suppressed locomotor activity in MPTP-injected mice, decreased number of central crossings, and elevated number of freezing events, suggesting increased anxiety in these groups of animals. Both treatments reduced the effects of MPTP on freezing behavior, as C_60_- and C_60_(OH)_24_-treated mice showed no significant changes in this behavior compared to controls. In addition, C_60_-treated mice had a higher number of central crossings than vehicle-treated MPTP mice did. Thus, both compounds exerted modest but significant ameliorative effects on anxiety-like behaviors in the MPTP model.

Physiological abnormalities in MPTP-injected mice are accompanied by profound neurochemical and molecular changes in the striatum and prefrontal cortex. Following MPTP exposure, striatal dopamine (DA) is progressively depleted, while its major metabolites, 3,4-dihydroxyphenylacetic acid (DOPAC) and homovanillic acid (HVA), decrease in parallel, reflecting impaired dopamine storage and turnover [81,82]. Levels of 3-methoxytyramine (3-MT), a marker of dopamine release into the extracellular space, showed a transient increase after MPTP treatment, indicating abnormal presynaptic release preceding terminal degeneration [82,83]. Interestingly, our study revealed an increase in striatal DA, whereas other parameters of DA release and turnover were consistent with previous observations. This can be explained by a shorter time window (12 h) between MPTP injection and HPLC evaluation of DA metabolism than that used in other studies (24 h and longer). Abnormal peaks in DA levels and presynaptic release in the striatum might be interpreted as a short compensatory response to neurotoxin administration that precedes the death of dopaminergic neurons [84]. While classical MPTP studies generally emphasize progressive striatal dopamine depletion measured 24 h post-injection, evidence suggests that surviving dopaminergic terminals can exhibit transient compensatory activity during the early post-injection period. These combined metabolic changes, acute DA release followed by chronic DA depletion, closely mirror the biphasic disruption of dopaminergic neurotransmission in human PD.

Notably, in the prefrontal cortex, DA levels and concentrations of DA metabolites were not significantly altered, but we found significant changes in the ratio of DOPAC/DA and DOPAC + DVA/DA, suggesting the involvement of this brain structure in PD-like symptoms, which is consistent with the literature [60]. Evidence indicates that projections from the prefrontal cortex can modulate striatal dopamine levels and enhance nigrostriatal dopamine activity [59]. Direct dopaminergic projections from the striatum to the prefrontal cortex are involved in the mechanisms underlying PD [58,59].

Here, antioxidant-treated animals that received a bolus injection of MPTP demonstrated partial preservation of dopaminergic markers in the striatum and the prefrontal cortex. The HPLC assay revealed rescue effects of both treatments on 3-MT in the striatum of MPTP-injected mice, suggesting ameliorated dopamine release into the extracellular space. Both fullerene and fullerenol normalized the DOPAC + DVA/DA ratio in the striatum and prefrontal cortex, suggesting an overall balancing effect of these compounds on PD-like neurochemical disturbances. Finally, regardless of the treatment conditions, we found increases in striatal serotonin in MPTP-injected mice that might manifest complex monoaminergic compensatory processes under conditions of acute neurotoxicity. This effect may be further enhanced by fullerene and fullerenol; other antioxidants have been shown to induce serotonin release in the brain [85].

In the prefrontal cortex, increased serotonin concentration was observed only in the fullerene-treated MPTP-injected group. It can be speculated that these effects of fullerene acting via the gut–brain axis [42] may overlap with the recently described peripheral mechanisms of stimulatory action of other antioxidants on serotonin release, whose effects are also mediated via modulation of the gut microbiota [86]. Reported monoaminergic MPTP-induced changes are accompanied by altered gene expression of several important regulators of dopamine turnover in the brain. There is a general effect of MPTP challenge on the gene expression of *nNOS* and *iNOS*, which govern oxidative stress and dopamine turnover in PD conditions [61,87]. No such increase was found in fullerenol-treated mice injected with MPTP, and fullerene-treated mice that received MPTP injection had unchanged *iNOS* as compared to controls. Thus, both compounds mitigated *iNOS* expression, suggesting that ameliorated nitrosative stress may be implicated in their effects on MPTP-challenged mice. The current study demonstrated that striatal *nNOS* expression was downregulated by fullerenol treatment but not by fullerene treatment. Fullerenol exerts superior antioxidant capabilities, enabling it to scavenge reactive nitrogen species (RNS), such as peroxynitrite (ONOO^−^) [88,89], a potent inducer of iNOS and nNOS expression [90]. Reduced RNS levels may lead to decreased activation of NF-κB and other transcription factors that upregulate iNOS and nNOS [91]. Expression of the gene encoding eNOS was not significantly altered by either manipulation. Indeed, it has been previously shown that fullerenol suppresses the activity and expression of NOS in the hippocampus [92]. Our study showed no changes in NOS expression in any form of the prefrontal cortex.

As discussed, MPTP administration increased the expression of *iNOS* and *nNOS,* which are often associated with oxidative and nitrosative stress contributing to neurodegeneration in PD [93]. Interestingly, fullerenol, but not fullerene, suppressed this upregulation, particularly for nNOS, which aligns with prior observations [41]. This distinction may reflect differences in bioavailability or tissue distribution, as fullerenol is water-soluble and may reach intracellular targets in the brain more readily. NO-related mechanisms have also been implicated in mitochondrial dysfunction and compromised PGC-1α expression, a factor of oxidative and nitrosative stress and dopaminergic neurodegeneration in PD patients and in MPTP models [69]. Most notably, we found that MPTP significantly affected expression of striatal *Pgc-1α*, an established regulator of mitochondrial biogenesis and function implicated in the pathology of PD [93]. The apparent normalization of *Pgc-1α* expression by fullerenol reinforces its potential role in preserving mitochondrial integrity under toxic conditions.

Since the neurodegenerative effects of MPTP were previously shown to be mediated via MAO-A and MAO-B, which mediate oxidative deamination, produce hydrogen peroxide, and thus exacerbate redox imbalance resulting from impaired dopamine metabolism [20,62,63], we studied the expression of genes encoding these molecules and found their significant change. Interestingly, the fullerenol-treated unchallenged group revealed an upregulation of both these genes, which might be interpreted as a sign of toxicity of this compound [94].

Previous studies have shown that decreased *Snca* mRNA levels in the substantia nigra and cortex may be indicative of the decompensation of regenerative processes in the early stages of PD and can also be induced by MPTP administration [95]. This study showed that, keeping with previous works, administration of MPTP had an overly suppressive effect on *Snca* expression [67], whereas the administration of antioxidants did not preclude this change. The expression of the gene encoding COMT, a marker in MPTP models [95], showed no significant changes in our study. Expression of the gene encoding TH, which catalyzes the rate-limiting step in dopamine synthesis, a marker of dopaminergic neuron loss [65,66], was markedly reduced in the striatum of MPTP-treated mice, with the exception of animals that received fullerenol C_60_(OH)_24_. The expression of synaptophysin, a marker of synaptic density [96], was unaltered by MPTP injection but was elevated in naïve mice dosed with fullerenol C_60_(OH)_24_. Notably, the brain expression of *Syp* can be compromised by oxidative stress and restored by antioxidants, such as coenzyme Q10 and lecithin [97]; previous studies showed its neuroprotective functions under conditions of brain insult [98]. Overall, gene expression changes provide supporting evidence for the validity of the MPTP model employed here as a paradigm of PD-like conditions. The protective effects of fullerene and fullerenol reported in this study are in line with previous in vitro and in vivo demonstrations of the potency of fullerenes in counteracting PD-related disease mechanisms [37,42].

The present study employed a regime of bolus MPTP administration. While chronic low-dose MPTP regimens are often considered more valid in mimicking PD by recapitulating gradual dopaminergic decline, neuroinflammation, and behavioral deterioration over time [99,100], acute regimens mimic the abrupt clinical onset following a long pre-symptomatic phase [101,102]. In this context, applied here acute MPTP bolus model may mimic the sudden loss of a critical neuronal population, sufficient to overcome compensatory mechanisms, thus providing clinical validity.

In our study, age-related hormonal and metabolic changes likely modulated both the toxicity of MPTP and the neuroprotective efficacy of fullerenes and fullerenols. Age-related physiological changes can alter drug disposition and pharmacodynamics, which can be due to the accumulation of lipophilic compounds due to increased adiposity [103] and reduced hepatic and renal drug clearance [104]. Perimenopausal estrogen decline modifies cytochrome activity and BBB integrity [105,106], potentially enhancing MPTP neurotoxicity while also increasing antioxidant accumulation [107,108]. Importantly, the doses of fullerene C_60_ and fullerenol C_60_(OH)_24_ used in our experiment were substantially lower that those applied in the most of in vivo studies, ranging from 460 μg/kg, i.p. [45], to 1 mg/kg [52] and 6.5 mg/kg fullerene C_60_ [42] and from 0.05 to 0.3 mg/kg/day for fullerenol C_60_(OH)_24_ [109]. Hence, our results support the hypothesis that low-dose antioxidant pretreatment, particularly with fullerene C_60_ and fullerenol C_60_(OH)_24_, can confer partial protection against MPTP-induced neurotoxicity, suggesting their potential utility in the treatment of PD.

However, our study had several limitations. It did not allow a direct comparison between sexes in the applied MPTP mode, and larger group sizes would likely help to increase statistical power. Next, we focused on acute effects within 90 min of MPTP challenge; longer-term studies are needed to assess whether these molecular changes translate into durable functional protection. The mechanisms of action of the two compounds, as well as histological effects and justification of gene expression changes at the protein level, were not addressed in our work, which is a limitation of the present study, while the abundant literature describes their pharmacokinetics and beneficial action on oxidative stress, nitric oxide signaling, dopaminergic neurotransmission, and mitochondrial function via peripheral or central mechanisms, as discussed above [37,42,47].

## 5. Conclusions

The present study demonstrated the validity of bolus administration of MPTP in aged female mice as a paradigm of PD that recapitulates the key neurochemical, behavioral, and molecular features of this disease, which are sensitive to a low dose of antioxidant treatments acting via distinct mechanisms. This offers the potential of this paradigm to screen for potential therapies and dietary interventions with antioxidant properties that act either via central or peripheral mechanisms in a rapid, cost-effective, and valid way. Apart from that, our results provide preliminary evidence of the potential of fullerene C_60_ and fullerenol C_60_(OH)_24_ to serve as drug candidates for PD pharmacotherapy.

## Data Availability

Data are available upon reasonable request. To access data, contact Tatyana Strekalova (tatslova@gmail.com).

## Supplementary Materials

The following supporting information can be downloaded at https://www.mdpi.com/article/10.3390/biomedicines13102425/s1, Table S1: Basic approaches to the application of modifications of PD models based on different modes of MPTP administration; Table S2: Sequences of the primers; Figure S1: Brain neurochemistry outcomes in the striatum following MPTP injection and dosing with C_60_ or C_60_(OH)_24_.

## Abbreviations

The following abbreviations are used in this manuscript

MPTP	1-Methyl-4-phenyl-1,2,3,6-tetrahydropyridine
PD	Parkinson’s disease
PCR	Polymerase chain reaction
NO	Nitric oxide
iNOS	Inducible nitric oxide synthase
nNOS	Neuronal nitric oxide synthase
eNOS	Endothelial nitric oxide synthase
PGC-1α	Peroxisome proliferator-activated receptor gamma coactivator 1-alpha
HPLC	High-pressure liquid chromatography
DA	Dopamine
DOPAC	3,4-Dihydroxyphenylacetic acid
HVA	Homovanillic acid
3-MT	3-Methoxytyramine
BBB	Blood–brain barrier
GFAP	Glial fibrillary acidic protein
IL	Interleukin
MAO-A	Monoamine oxidase A
MAO-B	Monoamine oxidase B
COMT	Catechol-O-methyltransferase
TH	Tyrosine hydroxylase
RNA	Ribonucleic acid
cDNA	Complementary deoxyribonucleic acid
GAPDH	Glyceraldehyde 3-phosphate dehydrogenase
ANOVA	Analysis of variance
CNS	Central nervous system
NRF2	Nuclear factor erythroid 2-related Factor 2
GCL	Glutamate–cysteine ligase
