# Supplementary material for: Bolus MPTP Injection in Aged Mice to Mimic Parkinson Disease: Effects of Low-Dose Antioxidant Treatment with Fullerene (C60) and Fullerenol (C60(OH)24)
Source: Biomedicines. 2025 Oct 3;13(10):2425. doi: 10.3390/biomedicines13102425 (PMC12561033; doi:10.3390/biomedicines13102425)
Supplement: Supplementary file 1 [file biomedicines-13-02425-s001.zip › biomedicines-3875283-supplementary.pdf]

Supplementary file

Supplementary Table S1. Basic approaches to the application of modifications of PD models based on different modes of MPTP administration

**Method of administration**	**Administration regimen (dose × frequency / duration)**	**Advantages**	**Disadvantages**	**Validity**
**Single acute administration [24]**	30 mg/kg ×1 (i/p) / 1 day	Ease of implementation, high reproducibility, possibility of rapid testing of potential drugs	Does not reproduce the progressive course of the disease	Suitable for primary screening and assessment of severe neurotoxic effects
**Multiple acute administration [26]**	20 mg/kg ×4 (i/p) / 1 day	Rapid induction of neurodegeneration, high reproducibility of results	Does not reflect the chronic course of the pathology, complicated application scheme compared to a single injection	Effective for studying the expressed pathophysiological mechanisms of PD
**Subacute administration [27]**	30 mg/kg ×5 (i/p) / 5 days	Modeling of early-stage neurodegeneration, moderate severity of symptoms	Less pronounced neuropathological changes compared to the acute model	Suitable for testing drug compounds with putative neuroprotective effects
**Chronic administration [28]**	30 mg/kg ×3 per week. / 4–5 weeks	Modeling the progressive course of the disease, the ability to track long-term changes	Long duration of the experiment, variability of responses	Applicable for studying the mechanisms of PD and for assessing the long-term effectiveness of therapies
**Low-dose chronic [25]**	4 mg/kg ×20 (i/p) / 20 days	High physiological relevance, similar to sporadic PD	Requires a long time, the effects of exposure are less pronounced, large scatter of data	Suitable for studying slowly developing pathological changes and mechanisms of PD
**Continuous infusion [29]**	0.2–0.3 mg/kg/h (SC, mini-pump) / 7–14 days	Constant toxin concentration, precise dosage control	Need for surgical implantation, high cost	It is used to simulate stable toxic effects simulating the development of PD
**Primate model [30]**	Individual mode	Maximum clinical comparability with human PD	Ethical restrictions, high cost, duration of the experiment	Used in translational research and preclinical studies of drug efficacy

Supplementary Table S2. Sequences of the primers

Gene	Forward sequence, 5′-3′	Reverse sequence, 3′-5′
Gapdh	TGCACCACCAACTGCTTAG	GGATGCAGGGATGATGTTC
Syp	TGCCAACAAGACGGAGAGTG	TAGTGCCCCCTTTAACGCAG
Mao-A	TCACAGGCCACATGTTCGAC	AACTCTATCCCGGGCTTCCA
Mao-B	CCACATTGACCAGACAGGGG	TCTTCATGCCCAAAGCAGGT
TH	GCCTCCTCACCTATGCACTC	CCCAGAGATGCAAGTCCAAT
Ppargc1a	GAATCAAGCCACTACAGACACCG	CATCCCTCTTGAGCCTTTCGTG
nNOS	CTGGTGAAGGAACGGGTCAG	CCGATCATTGACGGCGAGAAT
iNOS	GTTCTCAGCCCAACAATACAAGA	GTGGACGGGTCGATGTCAC
eNOS	CGAAGCGTGTGAAGGCAAC	TTGTACGGGCCTGACATTTCC
Snca	CTGCCCTTGCCTCTTTCATTG	TGAACACATCCATGGCTAAAGA
Comt	ATCCCAGGACCTTATCCCCC	GTGTCTGGAAGGTAGCGGTC

Significant effect of MPTP but not treatment or interaction was observed for the SncA expression in the striatum (F=5.138, p=0.0336; F=0.2328, p=0.7943 and F=0.4265, p=0.6581, respectively; two-way ANOVA; Suppl. Fig. S1A). No significant effects of MPTP, treatment, or their interaction were revealed for the Comt expression in the striatum (F=0.6177, p=0.4407; F=3.402, p=0.0525 and F=0.7584, p=0.4808, respectively; two-way ANOVA; Suppl. Fig. S1B).

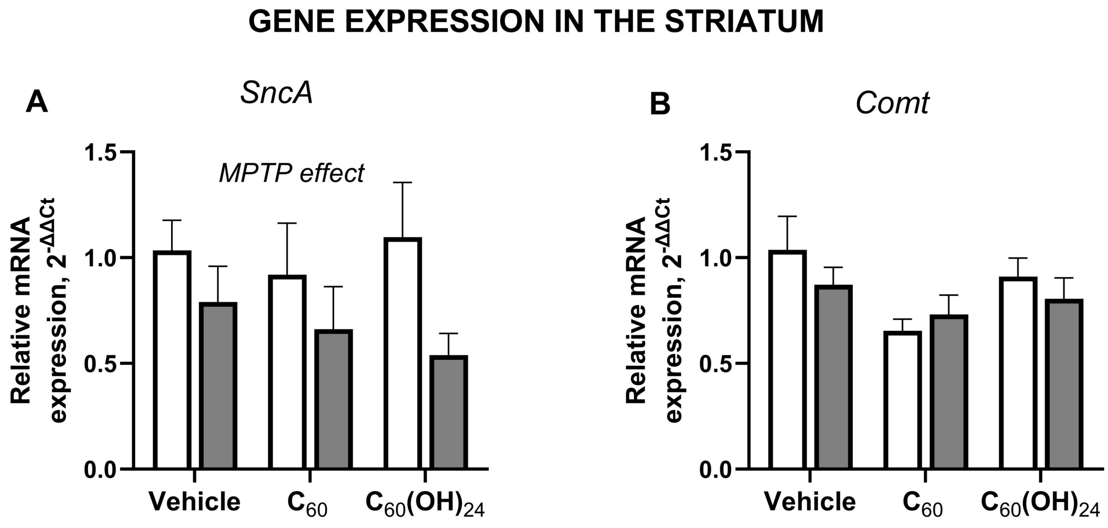

Supplementary Figure S1. Brain neurochemistry outcomes in the striatum following MPTP injection and dosing with C_{60} or $C_{60}(OH)_{24}$. (A) *SncA* expression was significantly decreased in MPTP-affected groups. **(B)** *Comt* expression was not affected by the MPTP injection. *p<0.05, two-way ANOVA with post hoc Tukey's test. N = 3-10 per group. Data is presented as mean ± SEM.

REFERENCES

1. Blesa J, Foffani G, Dehay B, Bezard E. Motor and non-motor circuit disturbances in early Parkinson disease: Which happens first? Nat Rev Neurosci. 2022, 23, 115–128. https://doi.org/10.1038/s41583-021-00533-y
2. Miguelez C, De Deurwaerdère P, Sgambato V. Editorial: Non-Dopaminergic Systems in Parkinson's Disease. Front Pharmacol. 2020, 11, 593822. https://doi.org/10.3389/fphar.2020.593822
3. Kim C. Non-motor symptoms of Parkinson's disease: Dopaminergic basis or not? Neurol Sci. 2019, 40, 2635–2636. https://doi.org/10.1007/s10072-019-04019-y
4. Dias V, Junn E, Mouradian MM. The role of oxidative stress in Parkinson's disease. J Parkinsons Dis. 2013, 3, 461–491. https://doi.org/10.3233/JPD-130230
5. Jávega-Cometto M, Naranjo-Viteri AJ, Champarini LG, Hereñú CB, Crespo R. Plant-Derived Monoterpene Therapies in Parkinson's Disease Models: Systematic Review and Meta-Analysis. Plants (Basel). 2025, 14, 999. https://doi.org/10.3390/plants14070999
6. Su CF, Jiang L, Zhang XW, Iyaswamy A, Li M. Resveratrol in Rodent Models of Parkinson's Disease: A Systematic Review of Experimental Studies. Front Pharmacol. 2021, 12, 644219. https://doi.org/10.3389/fphar.2021.644219
7. Zhang L, Dawson VL, Dawson TM. Role of nitric oxide in Parkinson's disease. Pharmacol Ther. 2006, 109, 33–41. https://doi.org/10.1016/j.pharmthera.2005.06.003
8. Gogna T, Housden BE, Houldsworth A. Exploring the role of reactive oxygen species in the pathogenesis and pathophysiology of Alzheimer's and Parkinson's disease and the efficacy of antioxidant treatment. Antioxidants (Basel). 2024, 13, 1138. https://doi.org/10.3390/antiox13091138
9. Talebi S, Ghoreishy SM, Jayedi A, Travica N, Mohammadi H. Dietary antioxidants and risk of Parkinson's disease: A systematic review and dose-response meta-analysis of observational studies. Adv Nutr. 2022, 13, 1493–1504. https://doi.org/10.1093/advances/nmac001
10. Koszła O, Stępnicki P, Zięba A, Grudzińska A, Matosiuk D, Kaczor AA. Current approaches and tools used in drug development against Parkinson's disease. Biomolecules. 2021, 11, 897. https://doi.org/10.3390/biom11060897
11. Dovonou A, Bolduc C, Soto Linan V, Leclerc L, Lévesque M, Hébert SS. Animal models of Parkinson's disease: Bridging the gap between disease hallmarks and research questions. Transl Neurodegener. 2023, 12, 36. https://doi.org/10.1186/s40035-023-00368-8
12. Chia SJ, Tan EK, Chao YX. Historical perspective: Models of Parkinson's disease. Int J Mol Sci. 2020, 21, 2464. https://doi.org/10.3390/ijms21072464
13. Khan E, Hasan I, Haque ME. Parkinson's disease: Exploring different animal model systems. Int J Mol Sci. 2023, 24, 9088. https://doi.org/10.3390/ijms24109088
14. Shadrina M, Slominsky P. Modeling Parkinson's disease: Not only rodents? Front Aging Neurosci. 2021, 13, 695718. https://doi.org/10.3389/fnagi.2021.695718
15. Machado V, Zöller T, Attaai A, Spittau B. Microglia-mediated neuroinflammation and neurotrophic factor-induced protection in the MPTP mouse model of Parkinson's disease—lessons from transgenic mice. Int J Mol Sci. 2016, 17, 151. https://doi.org/10.3390/ijms17020151
16. Ugalde-Muñiz P, Fetter-Pruneda I, Navarro L, García E, Chavarría A. Chronic systemic inflammation exacerbates neurotoxicity in a Parkinson's disease model. Oxid Med Cell Longev. 2020, 2020, 4807179. https://doi.org/10.1155/2020/4807179
17. Jackson-Lewis V, Przedborski S. Protocol for the MPTP mouse model of Parkinson's disease. Nat Protoc. 2007, 2, 141–151. https://doi.org/10.1038/nprot.2006.342

18. Tillerson JL, Miller GW. Detection of behavioral impairments correlated to neurochemical deficits in mice treated with moderate doses of 1-methyl-4-phenyl-1,2,3,6-tetrahydropyridine. Exp Neurol. 2002, 178, 80–90. https://doi.org/10.1006/exnr.2002.8007
19. Prasad EM, Hung SY. Behavioral tests in neurotoxin-induced animal models of Parkinson's disease. Antioxidants (Basel). 2020, 9, 1007. https://doi.org/10.3390/antiox9101007
20. Qiao C, Zhang Q, Jiang Q, Ma L, Tian Y, Wang Y. Inhibition of the hepatic Nlrp3 protects dopaminergic neurons via attenuating systemic inflammation in a MPTP/p mouse model of Parkinson's disease. J Neuroinflammation. 2018, 15, 193. https://doi.org/10.1186/s12974-018-1236-z
21. Klemann CJHM, Martens GJM, Poelmans G, Visser JE. Validity of the MPTP-treated mouse as a model for Parkinson's disease. Mol Neurobiol. 2016, 53, 1625–1636. https://doi.org/10.1007/s12035-015-9103-8
22. Masilamoni GJ, Smith Y. Chronic MPTP administration regimen in monkeys: A model of dopaminergic and non-dopaminergic cell loss in Parkinson's disease. J Neural Transm. 2017, 125, 337–363. https://doi.org/10.1007/s00702-017-1827-2
23. Bezard E, Dovero S, Imbert C, Boraud T, Gross CE, Bloch B. Effects of different schedules of MPTP administration on dopaminergic neurodegeneration in mice. Exp Neurol. 1997, 148, 288–292. https://doi.org/10.1006/exnr.1997.6632
24. Battaglia G, Busceti CL, Molinaro G, Biagioni F, Traficante A, Nicoletti F, Bruno V. Pharmacological activation of mGlu4 metabotropic glutamate receptors reduces nigrostriatal degeneration in mice treated with 1-methyl-4-phenyl-1,2,3,6-tetrahydropyridine. J Neurosci. 2006, 26, 7222–7229. https://doi.org/10.1523/JNEUROSCI.1595-06.2006
25. Schober A. Classic toxin-induced animal models of Parkinson's disease: 6-OHDA and MPTP. Cell Tissue Res. 2004, 318, 215–224. https://doi.org/10.1007/s00441-004-0938-y
26. Mustapha M, Taib CNM. MPTP-induced mouse model of Parkinson's disease: A promising direction for therapeutic interventions. Bosn J Basic Med Sci. 2021, 21, 422–433. https://doi.org/10.17305/bjbms.2020.4974
27. Ma Y, Rong Q. Effect of different MPTP administration intervals on mouse models of Parkinson's disease. Contrast Media Mol Imaging. 2022, 2022, 2112146. https://doi.org/10.1155/2022/2112146
28. Muñoz-Manchado AB, Villadiego J, Romo-Madero S, Bermejo-Navas A, Bermejo-Pareja F, López-Barneo J. Chronic and progressive Parkinson's disease MPTP model in adult and aged mice. J Neurochem. 2016, 136, 373–387. https://doi.org/10.1111/jnc.13409
29. Pain S, Gochard A, Bodard S, Gulhan Z, Prunier-Aesch C, Chalon S. Toxicity of MPTP on neurotransmission in three mouse models of Parkinson's disease. Exp Toxicol Pathol. 2013, 65, 689–694. https://doi.org/10.1016/j.etp.2012.09.001
30. Jenner P. The contribution of the MPTP-treated primate model to the development of new treatment strategies for Parkinson's disease. Parkinsonism Relat Disord. 2003, 9, 131–137. https://doi.org/10.1016/S1353-8020(02)00115-3
31. Dhillon AS, Tarbutton G, Levin JL, Kelley M, Kabadi S, Bach SB, et al. Pesticide/environmental exposures and Parkinson's disease in East Texas. J Agromedicine. 2008, 13, 37–48. https://doi.org/10.1080/10599240801986215
32. Nandipati S, Litvan I. Environmental exposures and Parkinson's disease. Int J Environ Res Public Health. 2016, 13, 881. https://doi.org/10.3390/ijerph13090881
33. Tat J, Heskett K, Boss GR. Acute rotenone poisoning: A scoping review. Heliyon. 2024, 10, e28334. https://doi.org/10.1016/j.heliyon.2024.e28334
34. Heikkila RE, Nicklas WJ, Vyas I, Duvoisin RC. Dopaminergic toxicity of rotenone and the 1-methyl-4-phenylpyridinium ion after their stereotaxic administration to rats. Neurosci Lett. 1985, 62, 389–394. https://doi.org/10.1016/0304-3940(85)90580-4

35. Greenamyre JT. Intersecting pathways to neurodegeneration in Parkinson's disease: Effects of the pesticide rotenone on DJ-1, alpha-synuclein, and the ubiquitin-proteasome system. Neurobiol Dis. 2006, 22, 404–420. https://doi.org/10.1016/j.nbd.2005.12.003
36. Sherer TB, Betarbet R, Testa CM, Seo BB, Richardson JR, Kim JH, et al. Mechanism of toxicity in rotenone models of Parkinson's disease. J Neurosci. 2003, 23, 10756–10764. https://doi.org/10.1523/JNEUROSCI.23-34-10756.2003
37. Miyazaki I, Asanuma M. The rotenone models reproducing central and peripheral features of Parkinson's disease. NeuroSci. 2020, 1, 1–14. https://doi.org/10.3390/neurosci1010001
38. Sherer TB, Kim JH, Betarbet R, Greenamyre JT. Subcutaneous rotenone exposure causes highly selective dopaminergic degeneration and alpha-synuclein aggregation. Exp Neurol. 2003, 179, 9–16. https://doi.org/10.1006/exnr.2002.8072
39. Cannon JR, Tapias VM, Na HM, Honick AS, Drolet RE, Greenamyre JT. A highly reproducible rotenone model of Parkinson's disease. Neurobiol Dis. 2009, 34, 279–290. https://doi.org/10.1016/j.nbd.2009.01.016
40. Sakai K, Gash DM. Effect of bilateral 6-OHDA lesions of the substantia nigra on locomotor activity in the rat. Brain Res. 1994, 633, 144–150. https://doi.org/10.1016/0006-8993(94)91533-4
41. Khaled R, Reichmann H, Gille G. Rotenone induces cell death in primary dopaminergic culture by increasing ROS production and inhibiting mitochondrial respiration. Neurochem Int. 2006, 49, 379–386. https://doi.org/10.1016/j.neuint.2006.02.002
42. Konnova E, Swanberg M. Animal models of Parkinson's disease. In: Parkinson's Disease: Pathogenesis and Clinical Aspects; Codon Publications: Brisbane, Australia, 2018; Chapter 5. https://doi.org/10.15586/codonpublications.parkinsonsdisease.2018.ch5
43. Zhang D, Li S, Hou L, Wang Z, Wang C, Liu Y, et al. Microglial activation contributes to cognitive impairments in rotenone-induced mouse Parkinson's disease model. J Neuroinflammation. 2021, 18, 4. https://doi.org/10.1186/s12974-020-02065-z
44. Tieu K. A guide to neurotoxic animal models of Parkinson's disease. Cold Spring Harb Perspect Med. 2011, 1, a009316. https://doi.org/10.1101/cshperspect.a009316
45. Ibarra-Gutiérrez MT, Serrano-García N, Orozco-Ibarra M. Rotenone-induced model of Parkinson's disease: Beyond mitochondrial complex I inhibition. Mol Neurobiol. 2023, 60, 1929–1948. https://doi.org/10.1007/s12035-022-03193-8
46. Tasselli M, Maggioli E, De Giorgio R, Blandizzi C, Fornai M. Effects of oral administration of rotenone on gastrointestinal functions in mice. Neurogastroenterol Motil. 2013, 25, e183–e193. https://doi.org/10.1111/nmo.12070
47. Riederer P, Müller T. Monoamine oxidase-B inhibitors in the treatment of Parkinson's disease: Clinical-pharmacological aspects. J Neural Transm. 2018, 125, 1751–1757. https://doi.org/10.1007/s00702-018-1876-2
48. Cuevas E, Burks S, Raymick J, Robinson B, Gómez-Crisóstomo NP, Escudero-Lourdes C, Sarkar S. Tauroursodeoxycholic acid (TUDCA) is neuroprotective in a chronic mouse model of Parkinson's disease. Nutr Neurosci. 2022, 25, 1374–1391. https://doi.org/10.1080/1028415X.2020.1859729
49. Meng T, Xiao D, Muhammed A, Deng J, Chen L, He J. Anti-inflammatory action and mechanisms of resveratrol. Molecules. 2021, 26, 229. https://doi.org/10.3390/molecules26010229
